# A Rare Case of Metachronous Quadruple Primary Malignancies in a Single Patient: A Case Report and Comprehensive Literature Review

**DOI:** 10.7759/cureus.25405

**Published:** 2022-05-27

**Authors:** Manisha Raikar, Shobha Mandal, FNU Manas, Victor O Kolade

**Affiliations:** 1 Internal Medicine, Guthrie Robert Packer Hospital, Sayre, USA; 2 Medicine, Guthrie Robert Packer Hospital, Sayre, USA

**Keywords:** multiple primary malignancies, clear cell renal cancer, adenocarcinoma lung, melanoma in-situ, chronic lymphocytic leukemia (cll)

## Abstract

Multiple primary malignancies (MPMs) in the same patient are rare. Over the past decade, the incidence of MPMs is increasing. The prevalence in the general population is 0.7-11.7%, with a higher incidence in the elderly. This increase in incidence can be attributed to advanced lifespan, environmental factors, early chronic disease/cancer screening, and advanced treatment leading to more metaplasia. The chances are higher in cancer patients due to the carcinogenic effect of chemoradiotherapy.

Here, we present a 79-year-old female with a 27 pack-year smoking history without any significant genetic predisposition, who developed four different primary malignancies including (1) chronic lymphocytic leukemia in 2017 (stage I modified Rai), positive for CD23 and CD5, which did not require treatment; (2) melanoma in situ on the left cheek in 2019 status post excision; (3) lung adenocarcinoma with negative molecular study (epidermal growth factor receptor (EGFR)/ROS proto-oncogene, receptor tyrosine kinase (ROS)/v-Raf murine sarcoma viral oncogene homolog B1 (BRAF)/anaplastic lymphoma kinase (ALK)) and negative programmed cell death ligand 1 (PDL-1) in 2020 for which she received treatment with carboplatin, pemetrexed, and pembrolizumab; and (4) left lower pole renal mass on surveillance CT scan, which was highly suspicious for primary malignancy as opposed to metastasis, for which she underwent radical nephrectomy and biopsy positive for clear cell renal cancer. Regarding these multiple primary cancers, the thought of germline mutation was considered. But as she did not have a family history of malignancy, genetic testing was not needed as per the genetic counselor.

Patients are being diagnosed with MPMs as there is more advancement in tumor detection and treatment. With the advancement in the treatment, cancer survivorship is improving. Given that there are no large studies, we believe that treatment modality for MPMs should be on a case-to-case basis and needs a multidisciplinary approach to tackle therapeutic challenges and provide radical treatment.

## Introduction

Multiple primary malignancies (MPMs) are described as more than one primary unrelated malignant lesion with different pathogenetic origins in an individual. The prevalence in the general population is 0.7-11.7% with a higher incidence in the elderly. Cancer survivors are at a 14% increased risk when compared to the general population to develop additional cancers [1]. Billroth first reported MPMs in a single patient in 1889 [2]. MPMs are confirmed by widely expected criteria proposed by Warren and Gates based on a thorough study on 1078 cancer autopsies [3]. The criteria are as follows: first, malignancies being histologically different from one another; second, anatomically separated by either in two different organs or, if present in the same organ, they should be separated by normal mucosa by at least 2 cm; and third, the chances of metastasis must be excluded. As this classification was overly broad, Moertel et al. classified based on multiple primary and multicentric malignancies, and also subclassified based on timing as synchronous (occurs simultaneously within six months) versus metachronous (one following the other after six months), with the latter being more common 2.7:1 ratio [4].

Since Warren and Gates released the criteria for diagnosing MPMs, many case reports have been published, but the occurrence of more than four cancers in the same patient is still a rare entity (<0.1%). The incidence of two primaries is up to 11% and the incidence decreases with an increase in number, and with four cancers, it is exceedingly rare [5]. We hereby present metachronous chronic lymphocytic leukemia (CLL), melanoma, lung adenocarcinoma, and clear cell renal cancer. These constellations of cancers have not been previously reported.

## Case presentation

**Figure 1:**
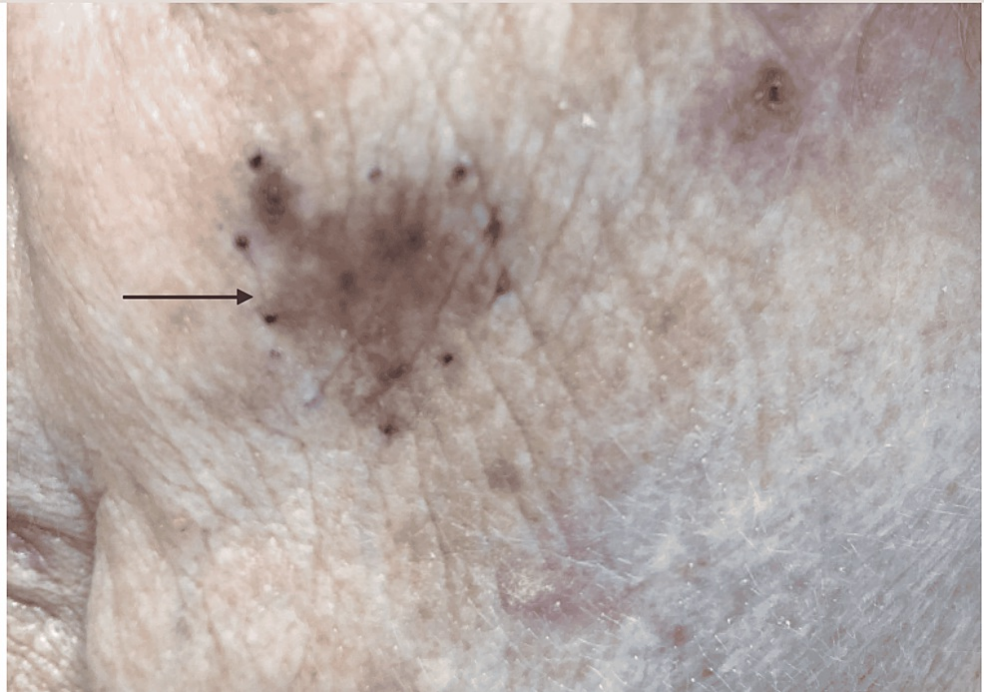
Dark pigmentation of the skin on the left cheek.

A 79-year-old female (gravida 2, para 2) with menarche at the age of 12 and menopause in her early 40s, on hormone replacement therapy for around seven years, with a 27 pack-year smoking history, active alcohol consumption of four drinks of whiskey per week, and a family history of unspecified lung cancer in her father was evaluated for lymphocytosis. At that time, flow cytometry confirmed CLL, which was positive for CD23 and CD5. She was asymptomatic and did not have any type B symptoms. She was diagnosed to have stage I modified Rai CLL; hence, she did not require treatment and was monitored. Two years later, she was diagnosed with melanoma in situ with a follicular extension on the left cheek with no ulceration or satellite lesions (Figure 1). Melanoma in situ was managed with excisional biopsy twice with more than 2 cm clear surgical margins. Melanoma in situ was of metachronous origin. She had multiple skin cancers including squamous cell cancer of the right thigh, nose, and left perineal region, and basal cell carcinoma on the right shoulder for which she underwent a Mohs procedure with clear margins.

**Figure 2:**
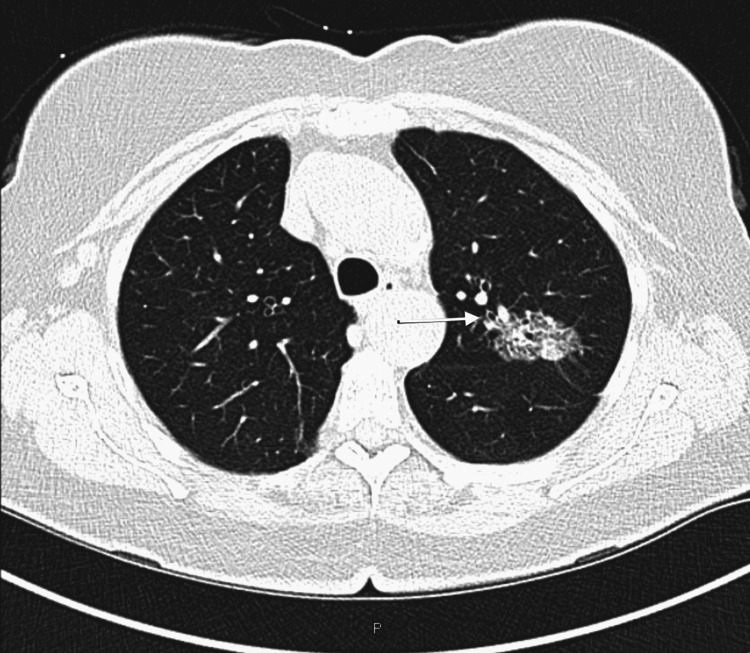
CT chest in the axial window shows a solid mass in the left upper lobe measuring 4.3 x 2 cm in axial dimensions and 3.9 cm in craniocaudal dimensions. The solid component measures 2.7 x 1.6 cm in axial dimensions and 1.7 cm in craniocaudal dimensions.

The patient was found to have a left upper lobe lung nodule (Figure 2), which had been followed over time with a repeat scan. As the pulmonary nodule was increasing in size, she underwent bronchoscopy with endobronchial ultrasound (EBUS) and transbronchial biopsy, which showed chronic inflammation. The mass was followed and continued to grow; hence, a positron emission tomography (PET)-CT scan was done, which showed fluorodeoxyglucose (FDG) avidity in the left upper lobe mass and bilateral uptake in mediastinal and hilar nodes (Figure 2). EBUS with biopsy of the mass, as well as lymph node sampling, was performed, with negative lymph nodes (atypical lymphocytes seen, consistent with known CLL), but the lung mass was positive for adenocarcinoma. Molecular testing could not be performed due to the sparsity of cells. It is uncertain if this was of synchronous or metachronous origin. She was initially considered to be at stage II of the disease, but a follow-up CT of the chest showed an increase in the size of the left upper lobe, and an increase in the size of multiple nodules in the left and right lung was noted, concerning for metastatic disease. She underwent a CT-guided biopsy of the left lower lobe lesion, which showed evidence of metastatic adenocarcinoma. The molecular study (epidermal growth factor receptor (EGFR)/ROS proto-oncogene, receptor tyrosine kinase (ROS)/v-Raf murine sarcoma viral oncogene homolog B1 (BRAF)/anaplastic lymphoma kinase (ALK)) was negative. Programmed cell death ligand 1 (PD-L1) expression was 0%. Lung cancer was treated with the regimen of carboplatin, pemetrexed, and pembrolizumab, and she received four cycles of treatment. A repeat CT scan of the lung showed a good response to therapy with a decrease in size of pulmonary masses initially but later showed metabolically active mass within the left upper lobe with a maximum standardized uptake value (SUV) of 13.6. She had stage IV adenocarcinoma in which she had T4 disease with lesions and separate ipsilateral lobe making her T4 N0 M1a stage IV adenocarcinoma. Pemetrexed was discontinued secondary to worsening anemia and she was continued on single maintenance therapy with pembrolizumab. She also received image-guided radiation therapy.

The patient was followed up on an outpatient basis with repeat imaging. A follow-up PET scan two years later showed a 3.8 cm solid-appearing lesion in the left lower pole of the kidney. She underwent a repeat CT scan of the abdomen and pelvis with contrast, which showed a 5.4 x 3.9 x 4.1 cm mass in the left kidney lower pole corresponding to the FDG-avid area. Urology suspected that this was primary kidney cancer as opposed to metastases of the lung. She underwent a robotic left radical nephrectomy given the good response of lung cancer to treatment. Postoperative pathological diagnosis confirmed clear renal cell carcinoma, which was synchronous in origin.

Regarding these multiple primary cancers, the thought of germline mutation was considered. But as she did not have a family history of malignancy, genetic testing was not needed as per the genetic counselor.

## Discussion

The pathogenesis for this entity is not fully elucidated at this point and is hypothesized to be from environmental factors like viruses, prolonged survival from advancement in health care, early detection from cancer screening and advancement in treatment modalities, and exposure to chemotherapy and ionizing radiation for primary malignancy [1]. A small study by Iioka et al. relates a possibility of “microsatellite instability” in the pathogenesis, which carries a worse prognosis when compared to sporadic cancers [6]. Another hypothesis suggested by Slaughter et al. is “field cancerization,” which means when one organ with cancer is exposed to a carcinogen, another organ without cancer is also exposed leading to a considerable risk of cancer, which in our patient could be related to lung and renal cancer with the exposure of smoking [7]. The “secondary Mullerian system” concept, which means cancer originates from a histologically similar organ, was suggested by Lauchlan et al., which here could be related to multiple skin malignancies (basal, squamous, and melanoma) originating from the epithelium [8]. In a study done by Warren and Gates, it was concluded that the incidence of MPMs is much higher than what would be expected as chance alone [3]. Some hypotheses regarding shared genes are associated with lung adenocarcinoma and renal cell carcinoma like Harvey rat sarcoma virus (HRAS) or P13KCA and TP53, which play a synchronous malignancy, but this needs further investigation [9].

## Conclusions

When there is an occurrence of a new mass, physicians must consider the possibility of new primary malignancy as a differential in a patient previously treated for cancer rather than assuming metastasis. As the incidence of MPMs is only case reports and no solid studies have yet been done, there are no established treatment guidelines. We believe that treatment modality for MPMs should be on a case-to-case basis based on types of malignancy, staging, and performance status. We need to have a multidisciplinary approach to tackle therapeutic challenges and provide radical treatment for a better outcome for the patient. Just because there are multiple malignancies does not infer poor prognosis and radical treatment is an option if it is a novel lesion and contained, detected, and treated early. The purpose of this review is to use this molecular mutation of undetermined significance for future reference for possible association with syndromes.
